# Understanding the key factors influencing knowledge management in business environments: a systematic review

**DOI:** 10.12688/f1000research.165390.1

**Published:** 2025-07-28

**Authors:** Muhammad Taufik Mardian, Mochammad Al Musadieq, Mohammad Iqbal, Ika Ruhana

**Affiliations:** 1 Faculty Of Administrative Sciences, Brawijaya University, Malang, East Java, 65145, Indonesia

**Keywords:** Knowledge Management, Leadership, Technology, Strategy, Human Resource, Innovation, Entrepreneurial

## Abstract

This study employs the PRISMA method to systematically explore knowledge management (KM) in business environments. A review of 44 selected studies identified eight key topics critical to enhancing KM: leadership, technology, strategy, human resource practices, high-performance work systems, innovation, entrepreneurial orientation, and learning orientation. These factors play essential roles in shaping effective KM practices. The findings show that leadership promotes collaboration and trust, creating a supportive environment for KM. Technological advancements facilitate efficient knowledge sharing and application, while strategic alignment ensures KM supports organizational goals. Human resource practices engage employees in knowledge processes, and high-performance work systems enhance knowledge acquisition and utilization. Innovation drives the generation of new ideas, and entrepreneurial orientation encourages a proactive approach to leveraging knowledge for competitive advantage. Despite the growing interest in KM, challenges such as resistance to change, inadequate infrastructure, and varying employee readiness hinder its effective implementation. This study emphasizes the need for an integrated KM approach that addresses these barriers while aligning KM strategies with organizational needs. Future research should investigate how these factors interact across different industries and cultural contexts to optimize KM practices and improve organizational performance.

## 1. Introduction

Knowledge management (KM) has become a critical determinant of organizational success in today’s dynamic and competitive business landscape. KM is the systematic process of acquiring, organizing, sharing, and applying knowledge to enhance organizational performance and innovation. As businesses increasingly recognize the value of knowledge as a strategic asset, the importance of KM in fostering adaptability, improving decision-making processes, and sustaining competitive advantage in rapidly changing environments has become more pronounced. ^1^ KM enables organizations to treat knowledge as a strategic resource. ^2, 3^ Traditionally, companies have prioritized tangible assets such as capital, labor, and raw materials. However, human resources, encompassing explicit knowledge (documented expertise, training programs, and organizational policies) and tacit knowledge (employee skills, experience, and insights), are now recognized as critical drivers of value creation. When effectively managed, this human capital can foster innovation, enhance problem-solving abilities, and strengthen decision-making processes, positioning organizations for sustained success. ^4– 6^

As organizations work to stay ahead of their competitors in an increasingly globalized and digital world, continuously accessing, sharing, and applying knowledge offers a distinct competitive edge. ^7^ A well-developed KM strategy allows companies to leverage their collective human resources, enabling faster responses to market changes, customer demands, and technological advancements. ^4, 5^ Furthermore, sharing best practices and lessons learned across departments or teams can help streamline operations, reduce redundancies, and improve overall efficiency. ^8^ In this way, KM becomes indispensable in navigating the constant changes that characterize today’s business environment. Economic shifts, technological disruptions, regulatory adjustments, and evolving customer expectations contribute to businesses’ unpredictability. ^9, 10^ Organizations that manage their knowledge effectively are better positioned to respond to these shifts, adapt to new circumstances, and capitalize on emerging opportunities.

KM frameworks that promote continuous learning, knowledge sharing, and cross-functional collaboration can enhance organizational agility and responsiveness. For example, businesses with strong KM practices are more likely to disseminate vital information to the right people at the right time, allowing quicker decision-making in uncertain environments. ^11^ This ability to transfer and apply knowledge quickly can mean the difference between seizing a market opportunity or falling behind competitors. Effective decision-making is crucial for organizational success, and KM plays a central role in ensuring that decisions are informed, data-driven, and aligned with long-term strategic goals. When employees at all levels have easy access to relevant and up-to-date knowledge, the quality of decisions improves. KM also enables organizations to identify patterns, insights, and best practices that may not be immediately obvious, helping reduce the risks associated with decisions made in isolation. ^12^ Moreover, by fostering collaboration across diverse teams, KM enables decision-makers to benefit from the collective intelligence within the organization, making for more holistic, informed decisions that lead to better outcomes.

Another critical aspect of KM is its role in fostering innovation, an essential driver of organizational growth and sustainability. Organizations must keep pace with industry trends and anticipate and lead change to remain competitive. ^13, 14^ KM helps create an environment conducive to innovation by supporting creativity and the free exchange of ideas. KM encourages employees to contribute their ideas, experiences, and expertise by promoting a knowledge-sharing and collaboration culture. ^15^ When knowledge is freely shared and integrated into the innovation process, organizations are better equipped to generate new solutions to complex problems and identify opportunities for product or service development. Moreover, KM systems that capture lessons from successes and failures provide valuable insights that help organizations refine their innovation strategies over time. ^14^

In modern business, competitive advantage is no longer solely about superior products or services. It is increasingly about superior knowledge. Organizations that systematically capture, store, and apply knowledge are better positioned to sustain long-term success. KM enables firms to preserve institutional memory, avoid redundancy, and improve operational efficiency. ^7^ By promoting continuous learning and improvement, KM also supports the development of expertise and thought leadership within an organization. ^16^ Over time, businesses with strong KM systems create a virtuous cycle in which continuous improvements in knowledge-sharing processes reinforce their competitive advantage, allowing them to stay ahead of the curve. ^3^ This cycle of knowledge-driven growth becomes difficult for competitors to replicate, especially in industries where expertise, innovation, and the ability to adapt quickly are the primary sources of differentiation.

Despite the undeniable importance of knowledge management (KM), its implementation often encounters significant challenges stemming from organizational, technological, and cultural factors. Issues such as resistance to change, inadequate infrastructure, lack of leadership support, and varying levels of employee readiness highlight the complexity of integrating KM into organizational processes. This underscores the need to comprehensively understand the drivers and barriers influencing its effectiveness. While KM has garnered substantial academic and practical attention, with studies exploring leadership styles, technological advancements, and external environmental factors, there remains a gap in synthesizing these findings to identify recurring themes, emerging trends, and areas requiring further investigation. Addressing this gap is essential for optimizing and tailoring KM strategies to meet the specific needs of diverse organizational contexts.

This study aims to bridge the gap by systematically reviewing the key factors influencing KM in business environments. By synthesizing empirical and theoretical research, the paper identifies critical success factors, common challenges, and practical implications for implementing KM initiatives. This review aims to provide valuable insights into the ongoing discourse on KM and establish a foundation for future research and practice. The structure of the paper is as follows: Section 1 provides a brief background; Section 2 discusses the relevant theories in this field; Section 3 outlines the information sources, study selection process, data collection methods, and data items; Section 4 presents the research findings; and finally, Section 5 summarizes the paper, highlighting its contributions, limitations, and suggestions for future research directions.

RQ: What critical factors influence the effectiveness of knowledge management practices in business environments?

## 2. Literature review

### 2.1 Knowledge management

Knowledge Management (KM) is a systematic approach to capturing, sharing, and utilizing organizational knowledge to drive innovation, improve efficiency, and gain competitive advantage. ^17^ Knowledge is considered a critical asset for organizations, encompassing best practices, decision-making processes, and creativity that are difficult for competitors to replicate. KM emerged to standardize creating, disseminating, and applying informational assets within businesses to promote growth and competitive edge. It involves the strategic management of intellectual resources, which is essential for creating value and fostering organizational success. ^2^ Research indicates that effective KM practices are integral to corporate sustainability, as organizations rely on acquiring and applying new knowledge to build core competencies and ensure long-term success. ^18^

Aligning knowledge management with organizational objectives supports sustainable growth and strengthens long-term competitiveness. Recent studies highlight the significant impact of KM capabilities on improving organizational performance. ^11^ Nonetheless, knowledge must be tailored to the organization’s unique context. Knowledge is recognized as an essential resource for generating value and maintaining competitive advantages in a volatile environment. ^19^ Additionally, the methods through which knowledge is generated and applied within companies are key, inimitable competencies that managers must identify and develop to foster lasting competitive advantages. ^20^ Effectively managing knowledge is vital for organizations aiming to stay competitive and sustainable in an ever-changing business environment. By aligning KM practices with organizational goals and adapting them to specific contexts, companies can enhance performance, create value, and maintain a long-term competitive edge.

Knowledge management is key in supporting organizational learning and adaptability by capturing insights from past experiences and promoting reflection and feedback. These processes enhance a firm’s resilience and agility, allowing it to thrive in dynamic environments. Additionally, KM helps preserve valuable knowledge assets in the face of technological changes, mitigating risks and ensuring their effective use to maintain a competitive edge. ^7^ To fully leverage the competitive advantages of information management, managers need to adopt supportive attitudes and a human-centered mindset, which can motivate employees to embrace new and existing knowledge. On the other hand, a closed-minded workforce and rigid management styles can undermine knowledge management efforts, ultimately stifling innovation and reducing the organization’s ability to adapt. ^21^ Successful knowledge management drives organizational learning, flexibility, and innovation. By fostering a culture of support and ensuring that valuable knowledge is preserved and applied effectively, organizations can adapt to changing environments and sustain their competitive edge.

### 2.2 Systematic literature review

A systematic literature review (SLR) is a methodical approach to research that involves gathering and evaluating studies on a specific topic. It serves various functions, such as identifying, reviewing, analyzing, and interpreting studies that address particular research questions. SLRs are often vital for shaping a research agenda, forming part of a dissertation or thesis, or assisting with research grant proposals. This method is widely used by researchers and scholars to review scientific literature, as it helps reduce bias and subjective interpretations of the results. SLR provides a comprehensive overview of research trends, their effectiveness, and the scope of earlier studies in a given field. ^22^

A systematic review adheres to established methodologies and guidelines for thoroughly searching, filtering, reviewing, evaluating, interpreting, synthesizing, and presenting findings from various publications on a specific subject. Its goal is to compile the most complete set of relevant literature, assess the quality of individual studies, and exclude those of low quality. With its comprehensive documentation of processes and assumptions, an SLR is designed to be replicable, often requiring a team of at least two people to minimize bias. Although time-consuming, the rigorous process of SLRs ensures they are a highly reliable source of evidence on a given topic. The PRISMA statement, introduced in 2009 by an international group of healthcare researchers, was developed to enhance the methodological rigor and quality of SLRs and meta-analyses. Initially intended for reviewing randomized trials, PRISMA has since been adapted to a range of research types. The systematic review process consists of several crucial steps: formulating the research question, forming a review team, defining search areas and sources, performing a systematic search, critically analyzing the data, interpreting the findings, and reporting the results. ^23^

## 3. Research method

### 3.1 Literature search strategy

The literature search was systematically designed to investigate and gather various comprehensive sources discussing knowledge management. This study uses the SLR approach suggested by. ^22^ Conducted in January 2025, the systematic literature review followed the Preferred Reporting Items for Systematic Reviews and Meta-Analysis (PRISMA) guidelines. A multi-database search strategy was employed, leveraging leading academic platforms such as Scopus. The search utilized keywords and phrases relevant to Knowledge Management.

**Figure 1.**
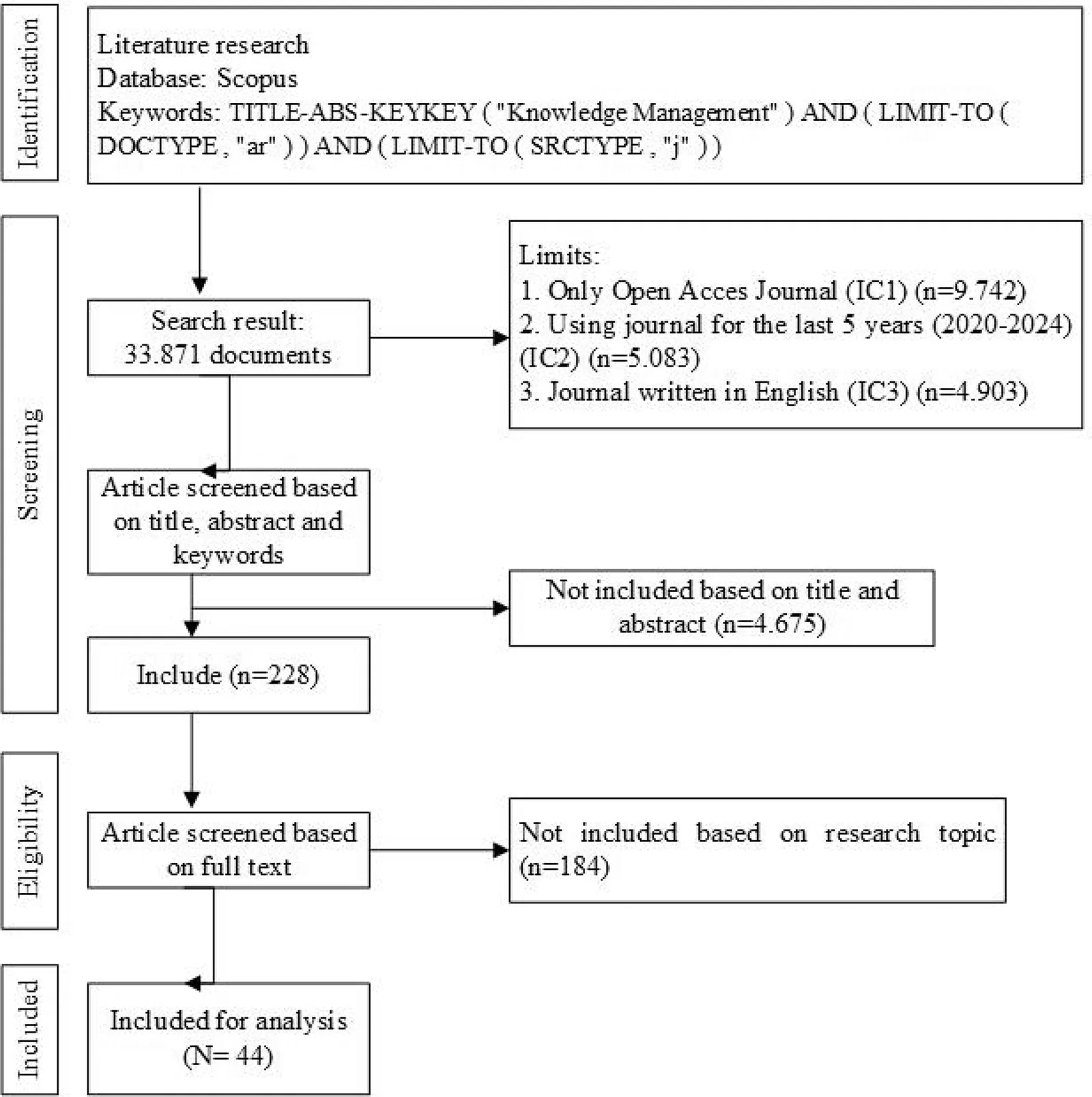
PRISMA Flow diagram.

The review targeted publications from 2020 to 2024 to ensure coverage of the most recent developments in Knowledge Management research. This approach seeks to provide a thorough and nuanced understanding of various perspectives and findings in the field. A PRISMA flow diagram was created and shown in Figure 1 to detail the article selection process.

### 3.2 Inclusion criteria

This study involved a manual article review process in four stages, including title, abstract, and full-text review. Relevant sources were defined through specific inclusion and exclusion criteria to guarantee their relevance and quality. The following inclusion criteria (IC) were used as guidelines for the systematic literature review to ensure the selection of relevant and high-quality studies:

IC1: Only open-access journal articles were considered to ensure the accessibility of full texts.

IC2: Journal articles published within the last five years (2020-2024) were included to prioritize recent and up-to-date research (excluded are books, book chapters, reviews, or conference reviews).

IC3: Only articles written in English were selected to maintain consistency in language and ensure the quality of the review.

IC4: Article containing empirical data, theoretical frameworks, and results regarding the determinants of knowledge management.

### 3.3 Information source

This study conducted an information search using online databases containing extensive collections of scholarly articles, such as Elsevier’s Scopus, which includes more than 25,000 peer-reviewed journals. Scopus was chosen as the primary source of information due to its broad coverage of research articles, ensuring a comprehensive representation of the existing literature on global consumer behavior. Articles not fully accessible were also excluded in this study to ensure that all selected studies were available for thorough analysis and evaluation.

### 3.4 Study selection

1) Using appropriate search keywords that align with the research objectives, focusing on Knowledge Management and related concepts. The search terms applied included TITLE-ABS-KEY (“Knowledge Management”) AND PUBYEAR > 2019 AND PUBYEAR < 2025 AND ( LIMIT-TO (DOCTYPE], “ar”)) AND ( LIMIT-TO (SRCTYPE, “j”)) AND (LIMIT-TO (OA, “all”)) AND (LIMIT-TO (LANGUAGE, “English”)).
2) Evaluating and selecting article titles, abstracts, and keywords in accordance with predefined eligibility criteria.
3) Carefully reviewing and selecting all articles that passed the previous phase, ensuring that they meet the eligibility criteria.

### 3.5 Data extraction and analysis

Key information, including authors, publication year, contextual scope, study objects, primary purpose, and recommendations, were systematically collected from the selected sources using a standardized approach. This comprehensive process ensured a structured analysis and data comparison across diverse studies. The synthesis of findings was designed to effectively address the research questions while considering geographical and contextual variations, thereby enhancing the relevance and applicability of the results to the research area.

## 4. Result and discussion

### 4.1 Result and qualitative synthesis

The research data from the SCOPUS database was retrieved using the following search terms: TITLE-ABS-KEY (“Knowledge Management”) AND (LIMIT-TO (DOCTYPE, “ar”)) AND (LIMIT-TO (SRCTYPE, “j”)). The initial search resulted in 33,871 articles. These articles were then filtered and selected based on the inclusion criteria specified in IC1, IC2, and IC3 by reviewing their titles, abstracts, and keywords. This screening process reduced the selection to 228 articles, which were fully reviewed. After completing this thorough selection, 44 articles were chosen for further examination.

From the 33.871 articles identified through Scopus, published between 2020 and 2024, these studies employed quantitative research approaches and explored various aspects of knowledge management across different countries and industries. A qualitative analysis was also conducted on the 44 selected articles, as shown in Table 1.

**Table 1.** Qualitative synthesis.

No	Author	Title	Object	Purpose
1	^24^	Driving Sustainable Performance in SMEs Through Frugal Innovation: The Nexus of Sustainable Leadership, Knowledge Management, and Dynamic Capabilities	236 craft SMEs in East Java, Indonesia	This study explores how sustainable leadership, dynamic capabilities, and knowledge management interplay with frugal innovation to enhance the sustainable performance of SMEs.
2	^17^	Big Data Analytics and Organizational Performance: Mediating Roles of Green Innovation and Knowledge Management in Telecommunications	384 management-level employees across five major telecom companies in Bangladesh	This study examines the impact of BDA on OP in Bangladesh’s telecommunications industry, with green innovation (GI) and knowledge management (KM) as mediating variables and big data analytics technical capabilities (BDATCs) as a moderating variable.
3	^25^	Effects of strategic leadership on change management: examining the mediating roles of accountability, knowledge management, and organizational culture in public organizations: a study in Central Gondar, Ethiopia	366 respondents in public organizations in Central Gondar, Ethiopia	This study aims to investigate the effects of strategic leadership on change management within public organizations, mediated by organizational culture, knowledge management, and accountability.
4	^26^	Enhancing Business Incubator Performances from Knowledge-Based Perspectives	100 technology business incubators operating throughout Indonesia	This study investigates 24 statement items aiming to uncover the factors influencing technology business incubators in Indonesia, focusing on knowledge management as a mediating variable from a knowledge-based perspective.
5	^14^	Sustainable innovation and business success: The mediating roles of information technology capability and knowledge management	180 SME in Saudi Arabia	In this study, our goal was to explore the connections between Sustainable Innovation (SI), Business Success (BS), Information Technology Capability (ITC), and Knowledge Management (KM) within Small and Medium Enterprises (SMEs) in Saudi Arabia.
6	^27^	The role of digital literacy and knowledge management on process innovation in SMEs	489 owner SME in Indonesia	This research investigates the effect of digital literacy on knowledge management, digital literacy on process innovation, and financial management on process innovation.
7	^13^	A mediated moderated analysis of knowledge management and stakeholder relationships between open innovation and performance of entrepreneurial firms	SME in Jordan	Current research attempts to address the gaps by analyzing the intricacies and dynamics of entrepreneurial firms’ involvement in Open Innovation.
8	^28^	Navigating Innovation Highways: Unraveling the Entrepreneurial Culture's Role with Knowledge Management as the Mediator in Automotive Technology Innovations	In five hundred questionnaires, the study received responses from 53 managers and 332 employees in e-vehicle automobile industries in India	This study aims to determine the impact of entrepreneurial Culture on technical innovation in the e-vehicle automobile business and examine the role of knowledge management as a mediator.
9	^6^	Role of entrepreneurial orientation, information management, and knowledge management in improving firm performance	150 small and medium-sized firms that manufacture furniture in Poland	The study aims to examine the causal relationship between IM and KM. Our sample consisted of 150 small and medium-sized firms manufacturing furniture in Poland.
10	^29^	The mediating role of knowledge management practices and balanced scorecard in the association between artificial intelligence and organization performance: evidence from MENA region commercial banks	594 employees working in commercial banks operated in the MENA region	Consequently, this research aims to answer these calls by providing and empirically testing a conceptual model that simultaneously takes into account Ai, KMP, and BSC with regard to their interactions with and effects on op.
11	^7^	Assessing the mediating role of knowledge management in the relationship between technological innovation and sustainable competitive advantage	370 respondents from 35 hotels	This study employed the resource-based view theory to investigate the relationships between technological innovation, knowledge management, and sustainable competitive advantage in the hospitality sector, focusing explicitly on hotels in the Yaounde and Douala regions of Cameroon.
12	^1^	Breaking down barriers: Exploring the impact of social capital on knowledge sharing and transfer in the workplace	30 information service firms in mainland China, resulting in 483 valid responses	This study delves into the influence of knowledge management processes on employees’ knowledge-sharing and transfer behaviors, viewed through the lens of the social exchange theory. It also probes the role of social capital in fostering and augmenting employees' involvement in refining these processes.
13	^11^	The analysis of a causal model of learning organization on sustainable organizational performance of public IT companies in China: Knowledge management practices and innovation capability as multiple mediators	46 employees from 10 public IT companies in China	This research aims to empirically examine the role of learning organization practices in enhancing sustainable organizational performance, utilizing knowledge management and innovation capability as mediating variables.
14	^30^	Analyzing the impact of excellence practices on organizational performance: Knowledge management as a mediator in mobile network operators	256 employees within four MnOs in Yemen	The purpose of this study is to analyze the impact of the eFQM excellence model in terms of the directional and execution excellence practices (the DePs and eePs) on organizational performance (OP). It also aims to analyze the mediation role of knowledge management processes (KMPs) between excellence practices and OP.
15	^31^	The influence of information technology, administrative management, and knowledge management practices on the success of e-government in Indonesia	380 questionnaires were distributed to respondents online via email and other online media DKI Jakarta province in implementing E-Government	This research aims to analyze the influence of information technology, administrative management, and knowledge management practices on the success of E-Government.
16	^32^	Organizational Integration, Knowledge Management, and Sustainable Entrepreneurship for SMEs in Developing Economies	490 SMEs Nigeria	This study underscores the indispensable role of knowledge management (KM) in promoting sustainable entrepreneurship (SE) among small and medium-sized enterprises (SMEs) in developing economies.
17	^2^	An empirical study of critical success factors in implementing knowledge management systems (KMS): The moderating role of culture	IT employees from different service sectors in the United Arab Emirates	This research focuses on the moderating effect of culture on the relationships between KMS and other variables affecting KMS in the service industry.
18	^15^	Driving intrapreneurial behavior through high-performance work systems	Employee banks in Ecuador	This paper analyzes the effect of employee perceptions of high-performance work systems on intrapreneurial behavior, with potential mediation by knowledge management processes.
19	^8^	Relationship between transformational leadership and knowledge management: The moderating effect of organizational culture	370 managerial staff members, both teaching and administrative, across various public universities in Peru	This study investigates how transformational leadership impacts knowledge management, focusing on the moderating effect of organizational culture.
20	^33^	Analyzing the effect of Corporate Social Responsibility on Green Innovation Performance in the Spanish wine industry: A structural equation modeling analysis	202 Spanish wineries	The present research analyses how Corporate Social Responsibility (CSR) practices affect Green Innovation Performance (GIP) in the Spanish wine industry.
21	^12^	Knowledge Management as Driver of Women’s Entrepreneurial Innovativeness	116 owner-managers SMEs in Bandung	The goal of this research is to assess the entrepreneurial traits of women business owners in Bandung, Indonesia, including innovativeness, knowledge management, e-commerce adoption, risk-taking, and technology optimism.
22	^34^	Does knowledge management mediate the relationship between entrepreneurial orientation and firm performance?	150 small furniture manufacturers operating in Poland	This study investigates the impact of entrepreneurial orientation (EO) and knowledge management (KM) on firm performance (PERF), as well as the mediating role of KM in the EO–PERF (EOPERF relationship).
23	^35^	Politics or markets: The dual role of the motivation to achieve organizational legitimacy in the development of knowledge management capabilities and business model innovation	236 Chinese new ventures running their businesses across a variety of sectors	We investigate how knowledge management capabilities affect business model innovation by exploring the dual role of different legitimation motivations in triggering knowledge management capabilities and moderating the relationship between knowledge management capabilities and business model innovation.
24	^4^	Sustainable human resource management practices in organizational performance: The mediating impacts of knowledge management and work engagement	500 respondents from the sampling frame of general Jordanian public university lecturers	This article presents a recent study outcome to examine (i) the mediating role of knowledge management and work engagement and (ii) the effect of sustainable HRM practices on organizational performance.
25	^36^	Clinical leadership and knowledge management: Essential role in patient safety culture?	237 nurses in Hospital “X” in West Nusa Tenggara	This study analyzes the influence of clinical leadership effectiveness on patient safety culture by distributing questionnaires to 237 nurses in Hospital “X” in West Nusa Tenggara.
26	^37^	Nurse Performance and Influence Factors in Discharge Planning Based on Knowledge Management SECI Model in Stroke Patients	133 stroke unit nurses at Jombang District Hospital, Ploso Hospital, and Jombang Hospital	This study aims to analyze the influence of nurse, family, patient, and organizational factors on the SECI Model knowledge management-based discharge planning in Jombang Regency, Indonesia.
27	^19^	Integrated e-learning for knowledge management and its impact on innovation performance among Jordanian manufacturing sector companies	57 Jordanian manufacturing companies were the study samples, and 470 managers were involved in this study at strategic, tactical, and operational levels	The present study investigated the relationship between knowledge management (KM) and innovation performance (IP). The mediating effect of knowledge Management was deeply explored.
28	^38^	Promoting intrapreneurial behavior in banking: The role of high-performance work systems, knowledge management processes, and supervisor support	Front line employees Bank in Ecuador	This paper analyzes the role of high-performance work systems, knowledge management processes, and supervisor support in promoting intrapreneurial behavior. The results indicate a positive and significant relationship between high-performance work systems and intrapreneurial behavior.
29	^39^	Investigating the Key Factors Influencing the Process Innovation Capability in Organizations: Evidence from the Republic of Serbia	264 Serbian companies that implemented ISO 9001 standard point to quality management	This study accentuates the importance of quality and knowledge management and their direct, mediating, and total effect on an organization’s process innovations.
30	^3^	The role of strategic agility towards competitiveness with mediating effect of knowledge management	Workers public higher education institution in Jordan	This study examines strategic agility's role in achieving competitiveness by analyzing the mediating effect of the knowledge management construct in Jordanian’s public higher education institutions.
31	^40^	The Impact of Employee Development Practices on Human Capital and Social Capital: The Mediating Contribution of Knowledge Management	464 employees working at information and communications technology companies	The current research aimed to consider the influence of employee development practices on intellectual capital through knowledge management.
32	^18^	Exploring the mediating role of knowledge management practices to corporate sustainability	130 respondents from nDhaka’s textile sector, a significant sector of Bangladesh’s economy and the world’s second-largest ready-made garment (RMG) manufacturer and exporter	This study aims to elaborate on and recognize a model in which knowledge management practices affect corporate sustainability via corporate structure, corporate culture, corporate leadership style, and a unique social capital variable used for the first time.
33	^5^	An Empirical Examination of Knowledge Management and Organizational Learning as Mediating Variables between HRM and Sustainable Organizational Performance	250 Questionnaires distributed to middle and top management levels in Thailand	Therefore, this article examines the mediating roles of knowledge management (KM) and organizational learning (OL) in the relationship between HRM and the long-term or sustainable performance of Thai construction firms.
34	^41^	The Effect of Digitalization on Innovation Capabilities through the Lenses of the Knowledge Management Strategy	620 Companies in Madrid	We proposed a comprehensive model that provides an integrative outlook on how the relationships between digitalization and knowledge management strategy predetermine the business results of the firm.
35	^20^	Knowledge management and intellectual capital in the business model innovation of Costa Rican manufacturing firms	100 Costa Rican manufacturing companies	This article analyzes the impact of knowledge management and intellectual capital on companies' ability to generate business model innovation.
36	^16^	The Effects of Transformational Leadership on Organizational Performance: Testing the Mediating Effects of Knowledge Management	1129 employees ini Cyprus Security Forces	The study analyzes the relationships between transformational leadership, knowledge management, organizational performance, job satisfaction, organizational learning, and knowledge creation processes.
37	^21^	The Relation among Organizational Culture, Knowledge Management, and Innovation Capability: Its Implication for Open Innovation	182 high-tech firm’s	This study was conducted to explore the relationship among organizational culture, knowledge management, and innovation capability in the open innovation environment and to provide useful suggestions and recommendations for managerial practices within the high-tech industry.
38	^42^	Examining the Link among Agility, Knowledge Management Practices and Firm Performance: Empirical Evidence from Electrical and Electronics Manufacturing Firms	85 manufacturing firms participated in this study	The study empirically investigates the relationship between agility and knowledge management practices on firm performance among Malaysia's electrical and electronics manufacturing firms.
39	^9^	Factors Affecting Knowledge Management and Its Effect on Organizational Performance: Mediating the Role of Human Capital	Steel plant in Afghanistan	This study aims to investigate and identify the factors affecting the empowerment and implementation of knowledge management in organizations and its impact on organizational performance.
40	^43^	Sustainability Performance of Organization: Mediating Role of Knowledge Management	99 respondents in Bali Province, Indonesia	This study examined the relationship between organizational culture and leadership styles with knowledge management and sustainable performance.
41	^44^	Effect of grit on the teaching creativity of Indonesian teachers: The mediating role of organizational commitment and knowledge management	496 Indonesian teachers located across four provinces (Jakarta, Banten, West Java, and Riau)	This study explores the effect of grit on the teaching creativity of Indonesian teachers, mediated by organizational commitment and knowledge management.
42	^45^	Unleashing the Importance of TQM and Knowledge Management for Organizational Sustainability in the Age of Circular Economy	manufacturing sector of a developing economy (n = 510)	The present research aims to examine the association between the constructs of total quality management (TQM) and organizational sustainability (OS) with the mediating effect of knowledge management (KM) from the perspective of a circular economy.
43	^46^	Impact of strategic capabilities on organizational ambidexterity in the commercial banks in Jordan: The mediating role of knowledge management	Manager banks in Jordan	This study seeks to explain the impact of strategic capabilities on organizational ambidexterity in the presence of knowledge management.
44	^10^	The Effective Factors on Knowledge Management in Universities from Physical Education Instructors' Viewpoints	all the instructors of physical education in Zanjan universities (61 people) with more than three years of teaching record	The present study aimed to investigate the relation of technology, Organizational culture and emotional intelligence with knowledge management using the mediators of organizational structure and empowerment.

**Figure 2.**
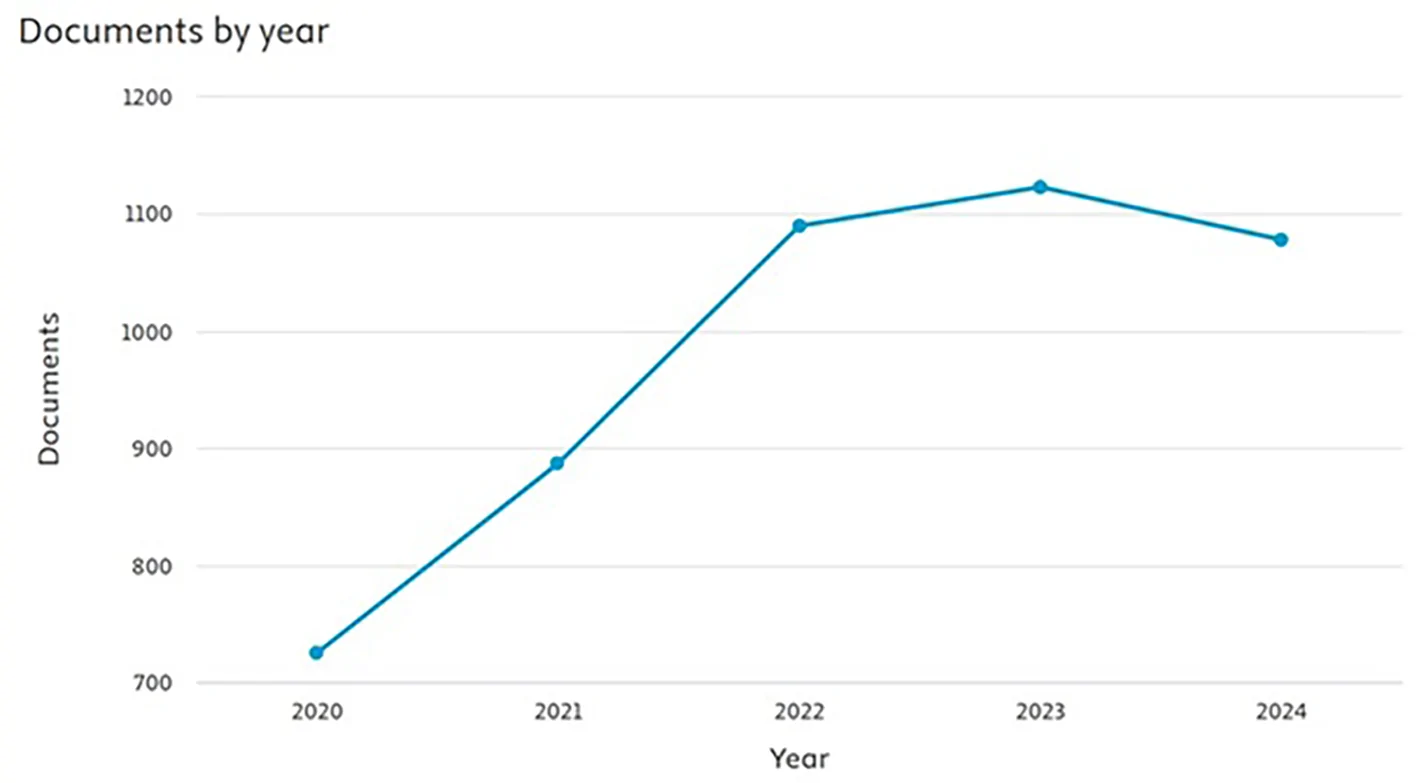
Distribution of selected studies over 5 years.

The chart in Figure 2 shows the progression in the number of publications focusing on knowledge management over the years. Between 2020 and 2022, the number of documents increased considerably, indicating a rising interest in knowledge management as a research area. This growth could be linked to heightened awareness of its role in supporting digital transformation and organizational adaptability. By 2023, the number of publications reached its peak, exceeding 1,100, showcasing the topic’s prominence and relevance during that year. However, a slight decline in the number of documents occurred in 2024, which may point to a shift in research priorities, the conclusion of significant studies, or natural variations in publication patterns. The chart reflects the growing importance of knowledge management as a research field, driven by organizational demands in an increasingly digital and dynamic environment.

### 4.2 Discussion

This systematic review explores the key factors influencing knowledge management in business environments, analyzing 44 articles published between 2020 and 2024. The studies reviewed cover various aspects of knowledge management, including methodologies, dimensions, and geographic contexts, offering a comprehensive understanding of its dynamics in organizational settings. The findings highlight the impact of leadership, technology, entrepreneurship, strategic orientation, learning orientation, human resources, high-performance work systems, and innovation on knowledge management practices. This review seeks to integrate findings from these studies to enhance theoretical models and offer actionable recommendations for improving knowledge management across various business contexts. The discussion will focus on the importance of each variable and explore the challenges associated with their application based on the insights gathered from the selected articles.

*4.2.1 Leadership*

The ten reviewed studies highlight that leadership is vital in enhancing knowledge management (KM) within organizations. Leadership styles, such as transformational, sustainable, clinical, and strategic leadership, drive KM practices, fostering innovation, improving performance, and contributing to organizational sustainability. For instance, Achmad and Wiratmadja ^24^ demonstrate how sustainable leadership enhances KM practices to support frugal innovation and long-term performance in SMEs. Similarly, Kılıç and Uludağ ^16^ and Sapta et al. ^43^ emphasize the significant role of transformational leadership in promoting knowledge sharing and creation, leading to improved performance in public institutions and traditional organizations. Furthermore, Al-Sous et al. ^19^ highlight the impact of knowledge-oriented leadership in fostering innovation performance through enhanced KM processes in Jordanian manufacturing firms.

Several studies also explore KM and organizational culture’s mediating and moderating roles in leadership-KM relationships. Ferede et al. ^25^ identify KM as a key mediator between strategic leadership and organizational change, emphasizing the importance of accountability and cultural integration. Similarly, Makiah et al. ^36^ show how clinical leadership in healthcare promotes patient safety culture by leveraging KM to facilitate knowledge sharing and organizational learning. Chung and Espinoza ^8^ further reveal that organizational culture strengthens the positive influence of transformational leadership on KM in academic institutions, highlighting the importance of a collaborative and adaptive culture. Additionally, studies such as Rezaei et al. ^9^ and Hossain et al. ^18^ demonstrate the role of KM in mediating leadership’s influence on sustainable performance, underscoring its critical importance in leveraging human capital and ensuring long-term organizational success.

These findings collectively underline the central role of leadership in fostering a knowledge-driven culture and optimizing KM practices. Leadership styles prioritizing collaboration, innovation, and accountability enable organizations to harness KM as a strategic tool for achieving competitive advantage, innovation, and sustainability. However, challenges such as resource constraints and resistance to knowledge sharing highlight the need for tailored leadership approaches and strategic investments in KM systems to maximize their impact. Leadership remains a cornerstone in driving effective knowledge management across various sectors, fostering collaboration, innovation, and adaptability. By addressing existing challenges and exploring new approaches, leadership can further optimize knowledge management practices, ultimately supporting organizational growth and long-term success in an ever-evolving global landscape.

*4.2.2 Technology*

Technology has emerged as a cornerstone in advancing knowledge management (KM) processes across diverse sectors, from education to government and private enterprises. Aljehani et al. ^17^ emphasize that integrating big data analytics (BDA) significantly enhances KM by enabling organizations to extract, store, and apply actionable insights that drive organizational performance. Similarly, Ramírez et al. ^41^ demonstrate the role of digitalization in fostering innovation capabilities through effective KM strategies, highlighting how digital tools optimize resource utilization and enhance decision-making processes. In the context of SMEs, Jasin et al. ^27^ reveal that digital literacy empowers employees to leverage KM for process innovation, suggesting that technology facilitates knowledge sharing and utilization at all organizational levels.

Moreover, advanced technologies such as artificial intelligence (AI) and knowledge management systems (KMS) further strengthen KM practices. Mahboub and Ghanem ^29^ show that AI enhances knowledge acquisition, distribution, and application, significantly boosting organizational performance in the banking sector. Abu-Alsondos ^2^ emphasizes the role of KMS in facilitating collaboration and knowledge-sharing activities, improving decision-making and operational efficiency when aligned with organizational strategies. Shafiee et al. ^10^ further elaborate on the importance of technology in supporting KM, illustrating its role in simplifying processes and ensuring knowledge accessibility within universities. These studies collectively underline the transformative role of technology in enhancing KM by enabling seamless communication, fostering collaboration, and driving innovation.

Several studies show that technology’s impact on KM extends to broader organizational outcomes. Susanto and Makmur ^31^ highlight how IT adoption in e-government frameworks improves public services and administrative efficiency while enabling robust KM practices. Rezaei et al. ^9^ explore KM implementation in Afghanistan’s Kabul Steel Plant, demonstrating that while technology positively influences KM, its success is amplified with leadership, organizational culture, and trust. In conclusion, integrating technology into knowledge management practices has proven to be a transformative force in driving innovation and organizational performance. By addressing challenges such as digital literacy gaps, resource limitations, and integration complexities, organizations can fully leverage technology’s potential to create a sustainable competitive advantage and adapt to the demands of an increasingly digital and knowledge-driven world.

*4.2.3 Entrepreneurial*

As the three reviewed studies highlighted, entrepreneurial orientation (EO) is crucial in shaping and enhancing organizational knowledge management (KM). These articles collectively demonstrate how entrepreneurial behaviors such as innovativeness, proactiveness, and risk-taking positively influence KM processes, which, in turn, contribute to improved firm performance and innovation. Sumathi and Padhy ^28^ highlight how an entrepreneurial culture in the electric vehicle sector in India drives technological innovation through KM, emphasizing the importance of knowledge acquisition, sharing, and utilization. Similarly, Kusa et al. ^34^ identify KM as a critical mediator between EO dimensions and firm performance, demonstrating how entrepreneurial behaviors promote knowledge creation and sharing as strategic resources for competitive advantage. Additionally, Kusa et al. ^6^ further reveal the causal relationship between EO and KM, showing that entrepreneurial efforts enhance KM practices and contribute to organizational growth and competitiveness.

The studies collectively underline the significance of integrating entrepreneurial behaviors with KM to unlock innovation and sustain competitive advantages. Entrepreneurial orientation encourages aligning KM practices with strategic decision-making, enabling firms to capitalize on opportunities and navigate market dynamics effectively. However, challenges such as limited resources, underdeveloped KM systems, and industry-specific constraints highlight the need for further research. Addressing these challenges will enable organizations to strengthen the synergy between entrepreneurship and KM, ensuring sustainable growth and innovation. Fostering a strong alignment between entrepreneurial orientation and knowledge management is essential for organizations to drive innovation, sustain competitive advantage, and adapt to dynamic market environments. By addressing existing challenges and leveraging entrepreneurial behaviors, firms can optimize knowledge processes for long-term growth and success.

*4.2.4 Strategic orientation and knowledge management*

Effective strategy is dynamic, adaptive, and rooted in clearly understanding an organization’s vision, mission, and objectives. A well-defined strategy ensures that KM is not an isolated function but an integrated component of the organization’s overall direction. This alignment allows organizations to capture, share, and apply knowledge to support innovation, enhance decision-making, and improve operational efficiency. Abazeed ^46^ demonstrates that strategic capabilities, such as marketing and management, significantly enhance KM by aligning internal resources with market opportunities, particularly in the banking sector. Similarly, Abuanzeh et al. ^3^ emphasize the impact of strategic agility on KM, showing that agility enables organizations to adapt to rapid market changes and improve their knowledge-sharing processes, ensuring competitiveness and sustainability. Abu-Alsondos ^2^ adds that a well-defined organizational strategy is essential for the success of knowledge management systems (KMS), as it ensures that KM practices are aligned with corporate objectives, fostering resource optimization, knowledge sharing, and innovation.

Rezaei et al. ^9^ further highlight the importance of aligning KM initiatives with strategic objectives to foster knowledge sharing, innovation, and organizational learning. KM efforts may lack direction without a coherent strategy, resulting in suboptimal outcomes. Collectively, these studies underscore that strategic integration is pivotal to the success of KM, ensuring its alignment with organizational goals and enhancing adaptability, innovation, and performance. Future research should explore the influence of different strategic frameworks on KM effectiveness across industries and cultural contexts to provide deeper insights into their interplay.

*4.2.5 Learning orientation and knowledge management*

Qin et al. ^11^ emphasize that fostering a continuous organizational learning culture significantly improves KM practices, particularly in the Chinese IT sector. By embedding learning-oriented practices, organizations can optimize knowledge sharing, storage, and application, which is essential for navigating rapid technological changes and achieving sustainable performance. Similarly, Al-Sous et al. ^19^ highlight the importance of integrating e-learning systems with KM in Jordanian manufacturing companies. Their findings show that e-learning enhances knowledge transfer, storage, and utilization as a strategic component of organizational learning, creating a dynamic environment that supports KM processes and improves organizational performance. Together, these studies underscore that cultivating a strong learning culture and leveraging technology-enabled learning systems are crucial for strengthening KM frameworks and ensuring adaptability and competitiveness in dynamic environments.

*4.2.6 Innovation and knowledge management*

Innovation is deeply interconnected with the effective management of knowledge within an organization. KM facilitates innovation by providing the tools and processes to capture, share, and apply knowledge. This includes leveraging organizational knowledge to generate new ideas, fostering collaboration, and integrating knowledge into decision-making processes. Hussien et al. ^14^ highlight that sustainable innovation fosters KM by improving organizational processes and integrating innovative practices that align with knowledge management systems. Their study on SMEs in Saudi Arabia demonstrates how sustainable innovation supports the development of IT capabilities and KM practices, contributing to improved business performance. Similarly, Kanaan et al. ^13^ emphasize the role of open innovation in entrepreneurial firms, illustrating that KM mediates the relationship between open innovation and organizational performance. This mediation demonstrates KM’s pivotal role in utilizing innovation to enhance stakeholder relationships and achieve competitive advantages.

Nyuga and Tanova ^7^ provide additional insights, focusing on the hospitality industry in Cameroon. Their study shows how technological innovation positively influences KM by enabling efficient collection, organization, and information sharing. When paired with technological advancements, they found that KM systems significantly enhance operational efficiency and service quality, contributing to sustainable competitive advantages. Organizations can better adapt to market demands and technological changesby fostering a culture of innovation and knowledge exchange.

These studies collectively demonstrate the integral role of innovation in enhancing KM practices. Sustainable and technological innovations enable organizations to optimize KM systems, fostering a culture of continuous learning and adaptation. By leveraging innovation, organizations can effectively utilize knowledge, thus gaining a competitive edge and driving long-term success.

*4.2.7 Human resource*

HR functions such as recruitment, training, performance management, and employee development are critical in fostering a culture of knowledge sharing and utilization. By hiring individuals with the right skills and fostering continuous learning, organizations create an environment where knowledge can be effectively acquired, shared, and applied. The study by Kokkaew et al. ^5^ highlights that effective human resource management (HRM) practices, when aligned with organizational learning, significantly enhance the processes of knowledge acquisition, sharing, and utilization in the Thai construction industry. They emphasize that the fragmented nature of construction work poses challenges to KM. However, HRM practices, such as selective recruitment and employee development, help overcome these barriers by fostering an environment conducive to learning and innovation.

Similarly, Abu-Mahfouz et al. ^4^ argue that sustainable HRM practices are essential for optimizing KM systems in higher education institutions in Jordan. Their findings reveal that practices like training, employee engagement, and structured development programs enhance KM’s effectiveness and contribute to organizational performance. By integrating KM with HRM strategies, institutions can achieve greater collaboration, knowledge sharing, and innovation, reinforcing their competitive advantage in dynamic environments.

Both studies highlight the essential interplay between HRM practices and knowledge management (KM), emphasizing that robust HRM systems are a foundation for effective KM implementation. Organizations should prioritize sustainable and learning-oriented HR practices to cultivate a knowledge-driven culture that fosters long-term growth and adaptability in an ever-evolving business landscape. A strong focus on employee engagement and motivation ensures the seamless flow of knowledge across departments, enhancing organizational performance and competitiveness. Integrating HR practices with KM strategies ultimately empowers organizations to establish a sustainable, knowledge-centered culture that drives innovation, resilience, and sustained success.

*4.2.8 High-performance work systems*

High-performance work systems (HPWS) refer to interrelated human resource management (HRM) practices designed to maximize employee performance, engagement, and contribution to organizational goals. These systems typically include practices such as selective recruitment, comprehensive training, performance-based rewards, employee empowerment, and participative decision-making. By equipping employees with the necessary skills and providing opportunities for collaboration and autonomy, HPWS enables effective knowledge sharing, acquisition, and utilization. The study by Portalanza-Chavarría and Revuelto-Taboada ^15^ highlight that HPWS is pivotal in fostering knowledge-sharing processes, enhancing employee capabilities, and facilitating collaboration. These systems provide employees the necessary resources, training, and autonomy to participate actively in KM processes. By empowering employees through targeted HR practices, HPWS encourages acquiring, sharing, and applying knowledge, which is essential for organizational learning and innovation.

Similarly, Revuelto-Taboada et al. ^38^ emphasize that HPWS are instrumental in promoting a knowledge-driven culture, particularly in knowledge-intensive industries like banking. Their findings suggest that HPWS creates an environment conducive to effective KM by aligning employee behaviors with organizational goals. HPWS facilitates knowledge acquisition and interpretation processes, enabling organizations to adapt to dynamic market conditions and maintain a competitive edge. These systems enhance individual and team knowledge-sharing behaviors and contribute to building a sustainable framework for continuous learning and innovation.

Both studies underscore the importance of HPWS in driving KM processes. By integrating robust HR practices into their strategies, organizations can establish a strong foundation for knowledge management, ensuring adaptability and long-term success in an ever-changing business environment. HPWS is a critical enabler for fostering a knowledge-centered culture and enhancing organizational resilience and innovation capacity.

## 5. Conclusion

The discussions emphasize the pivotal role of Knowledge Management (KM) as a foundational element for organizational success. KM bridges critical organizational components such as Leadership, Technology, Strategy, Human Resource Practices, High-Performance Work Systems, Innovation, and Entrepreneurial Orientation. Effective leadership fosters collaboration and trust, creating an environment conducive to KM. Technology enables efficient knowledge-sharing and utilization through advanced tools and systems like artificial intelligence and big data analytics. The strategy ensures that KM aligns with organizational goals, facilitating integration into decision-making processes. Human resource practices and high-performance work systems empower employees to contribute to KM actively. At the same time, innovation and entrepreneurial orientation provide a dynamic framework for leveraging knowledge to seize opportunities and drive competitiveness. Collectively, these factors underscore KM’s centrality in fostering innovation, adaptability, and long-term organizational performance.

The findings suggest organizations must integrate KM into their core strategies to optimize their potential. This requires aligning KM practices with leadership initiatives, investing in technology to enhance knowledge-sharing processes, and fostering a culture that values continuous learning and collaboration. Policymakers and industry leaders should also support KM adoption by promoting access to digital infrastructure, providing training programs to improve organizational capabilities, and encouraging cross-industry knowledge-sharing initiatives. Organizations prioritizing KM will be better equipped to adapt to rapidly changing environments, innovate effectively, and maintain a competitive advantage.

Future research should explore the evolving dynamics between KM and emerging leadership styles, such as digital, servant, and agile leadership, particularly in diverse cultural and industrial contexts. Additionally, integrating advanced technologies like blockchain and machine learning into KM systems warrants further investigation, as does the alignment of KM with strategic objectives during periods of uncertainty or disruption. Research on the role of human resource practices in supporting KM, especially in fostering sustainable and diversity-focused environments, remains critical. Moreover, understanding how KM mediates the relationship between entrepreneurial orientation and innovation across different sectors and regions could provide valuable insights into enhancing organizational resilience and adaptability. Comparative studies focusing on KM practices across industries and regions will also contribute to a more comprehensive understanding of its challenges and opportunities. These directions will strengthen KM as a cornerstone for innovation, growth, and sustainability in an increasingly complex global landscape.

## Ethics and consent

Ethical approval and consent were not required.

## Data Availability

The data generated and/or analyzed during this study are available in the [figshare] repository, under the open license [CC-BY or CC0]. The data can be accessed via the following link: [ https://figshare.com/s/b942ac704f9726110ad3] & [ https://figshare.com/s/c7b55251c46c1fb15eb0], and the DOI assigned to this data is [10.6084/m9.figshare.29467184] & [10.6084/m9.figshare.29467181]. ^47^ The supplementary data for this study can be accessed via the following link: [ https://figshare.com/s/399ace89813b0ecb992f], and the corresponding DOI is [10.6084/m9.figshare.29467973]. ^47^ Data are available under the terms of the Creative Commons Attribution 4.0 International license (CC-BY 4.0).
