# Supplementary material for: Understanding the key factors influencing knowledge management in business environments: a systematic review
Source: F1000Res. 2026 Jul 10;14:738. Originally published 2025 Jul 28. [Version 4] doi: 10.12688/f1000research.165390.4 (PMC13585040; doi:10.12688/f1000research.165390.4)
Supplement: Peer review history

# Reviewer report: Reviewer response for version 4

**DOI:** 10.5256/f1000research.205354.r502226

**Published:** 2026-07-20

**Authors:** Eka Desy Purnama

**Affiliations:** 1 Krida Wacana Christian University, Jakarta, Jakarta, Indonesia

The current version of the manuscript is generally clear, well organized, and methodologically sound. The systematic review process is explained with sufficient transparency, while the findings, conceptual framework, theoretical implications, and limitations are presented in a coherent manner. The revisions made across the previous versions have also strengthened the overall quality and transparency of the manuscript.

**Minor Comments**

1. Some key terms related to knowledge management, organizational performance, innovation, and contextual conditions are used somewhat interchangeably across different sections. The authors are encouraged to ensure that each term is used consistently according to its intended meaning.
2. Some parts of the discussion repeat similar points regarding the contributions of leadership, technology, human capabilities, and innovation to knowledge management. Minor editing to reduce repetition would make the discussion more concise and focused.
3. Minor editorial inconsistencies, including capitalization, numerical formatting, and the use of abbreviations, should be reviewed throughout the manuscript.

**Final assessment**

 The remaining concern is editorial and does not affect the reliability of the findings or the overall contribution of the study. The manuscript is therefore assessed as **Approved**, with minor recommendations to improve clarity and consistency.

Are the rationale for, and objectives of, the Systematic Review clearly stated?

Yes

Is the statistical analysis and its interpretation appropriate?

Not applicable

If this is a Living Systematic Review, is the ‘living’ method appropriate and is the search schedule clearly defined and justified? (‘Living Systematic Review’ or a variation of this term should be included in the title.)

Not applicable

Are sufficient details of the methods and analysis provided to allow replication by others?

Yes

Are the conclusions drawn adequately supported by the results presented in the review?

Yes

Reviewer Expertise:

Business Administration; Management; Business and Economics

I confirm that I have read this submission and believe that I have an appropriate level of expertise to confirm that it is of an acceptable scientific standard.

# Reviewer report: Reviewer response for version 3

**DOI:** 10.5256/f1000research.200471.r486333

**Published:** 2026-06-15

**Authors:** Hellen Ogutu

**Affiliations:** 1 Doctoral School of Economics and Regional Science,, Godollo, Hungary

***Decision: Minor Revision***

 The revised manuscript is substantially stronger than previous versions and demonstrates clear effort in responding to reviewer feedback. The introduction, methodology, conceptual framework, implications, and conclusion sections have all improved. The proposed framework represents the manuscript's principal contribution and provides a useful basis for future KM research. Before indexing, the paper would benefit from deeper critical synthesis of the reviewed studies, stronger theoretical anchoring, discussion of study quality and research biases, and minor language and formatting corrections. Subject to these revisions, the manuscript is suitable for indexing.

**Review Comments and *Recommendation to the Authors***

***Decision: Minor Revision***

 The manuscript has improved considerably and now makes a meaningful contribution to the KM literature through its integrative framework.

 The major concerns raised in earlier reviews have largely been addressed.

 However, manuscript would benefit from one final round of revision focused on:

1. *Increasing critical synthesis rather than description.*
2. *Discussing methodological weaknesses of reviewed studies.*
3. *Acknowledging the absence of quality appraisal.*
4. *Strengthening theoretical grounding.*
5. *Correcting remaining language and formatting issues.*

*Provided these issues are addressed satisfactorily, the manuscript would be suitable for indexing.*

Are the rationale for, and objectives of, the Systematic Review clearly stated?

Partly

Is the statistical analysis and its interpretation appropriate?

Partly

If this is a Living Systematic Review, is the ‘living’ method appropriate and is the search schedule clearly defined and justified? (‘Living Systematic Review’ or a variation of this term should be included in the title.)

Not applicable

Are sufficient details of the methods and analysis provided to allow replication by others?

Partly

Are the conclusions drawn adequately supported by the results presented in the review?

Partly

Reviewer Expertise:

Knowledge Management and Sustainability

I confirm that I have read this submission and believe that I have an appropriate level of expertise to confirm that it is of an acceptable scientific standard, however I have significant reservations, as outlined above.

# Response

**Published:** 2026-07-01

**Authors:** Muhammad Taufik Mardian

**Affiliations:** Faculty of Administrative Scie, Brawijaya University, Malang, East Java, Indonesia

Thank you for the constructive comments and positive assessment of our revised manuscript. In response to the remaining suggestions, we have revised the manuscript as follows.

 1. Increasing critical synthesis rather than description.

**Author's response:**  We strengthened the Discussion section by adding a critical synthesis that explains the identified KM factors as interrelated conditions rather than isolated determinants. We also clarified the dual role of KM as both an outcome and a mediating mechanism.

 2. Discussing methodological weaknesses of reviewed studies.

**Author's response:**  We added a discussion of methodological weaknesses across the reviewed studies, including the frequent use of cross-sectional designs, self-reported data, and context-specific samples, which may limit causal inference, increase common method bias, and affect generalizability.

 3. Acknowledging the absence of quality appraisal.

**Author's response:** We explicitly acknowledged in the Limitations section that no formal quality appraisal or risk-of-bias assessment was conducted and noted that variations in study quality may affect the robustness of the synthesis.

 4. Strengthening theoretical grounding.

**Author's response:**  We strengthened the theoretical grounding of the framework by improving its conceptual clarity and coherence.

 5. Correcting remaining language and formatting issues.

**Author's response:**  We revised the manuscript for clarity, consistency, language, and formatting, including removing repetition, standardizing terminology, correcting typographical errors, and checking headings, tables, figures, and references.

 We hope that the revisions satisfactorily address the reviewer’s concerns and further strengthen the manuscript’s contribution to the knowledge management literature. We are grateful for the reviewer’s constructive comments and valuable suggestions.

# Reviewer report: Reviewer response for version 2

**DOI:** 10.5256/f1000research.193303.r443025

**Published:** 2026-01-08

**Authors:** Hellen Ogutu

**Affiliations:** 1 Doctoral School of Economics and Regional Science,, Godollo, Hungary

**Summary of the reviewer’s evaluation**

- The manuscript addresses a **relevant topic.** However, the review is **largely descriptive**. It identifies key KM factors but does not sufficiently explain **what is new** compared to existing KM reviews, nor does it integrate the findings through a clear **theoretical lens**.
- The research question is broad, and the conceptual framework requires stronger theoretical justification.
- The **search strategy is narrow** (single database and keyword), and the **exclusion criteria, **especially the exclusion of review articles, are weakly justified. These choices limit coverage and raise concerns about selection bias.
- The **discussion and conclusions** summarize findings, but lack deeper **analytical synthesis**, cross-study comparison, and explanation of relationships among factors.

**Overall recommendation:**

 ✔ **Not Approved**

 The paper is indexable in principle, but it requires **(very) major revisions, a** stronger theoretical integration, clearer articulation of contribution, deeper analytical synthesis, and more critical reflection on methodological choices to reach its full scholarly potential.

Are the rationale for, and objectives of, the Systematic Review clearly stated?

Partly

Is the statistical analysis and its interpretation appropriate?

Partly

If this is a Living Systematic Review, is the ‘living’ method appropriate and is the search schedule clearly defined and justified? (‘Living Systematic Review’ or a variation of this term should be included in the title.)

Not applicable

Are sufficient details of the methods and analysis provided to allow replication by others?

Partly

Are the conclusions drawn adequately supported by the results presented in the review?

Partly

Reviewer Expertise:

Knowledge Management and Sustainability

I confirm that I have read this submission and believe that I have an appropriate level of expertise to state that I do not consider it to be of an acceptable scientific standard, for reasons outlined above.

# Response

**Published:** 2026-04-27

**Authors:** Muhammad Taufik Mardian

**Affiliations:** Faculty of Administrative Scie, Brawijaya University, Malang, East Java, Indonesia

Thank you for this valuable comment. We agree that the previous version was largely descriptive and did not sufficiently clarify the novelty of the review. In response, we revised the Introduction to explain the novelty more clearly. The manuscript now emphasizes that the review does not only identify KM-related factors, but also organizes them into an integrative conceptual framework. This revision helps move the manuscript beyond a descriptive list of variables and provides a clearer conceptual contribution.

 1. The manuscript addresses a relevant topic. However, the review is largely descriptive. It identifies key KM factors but does not sufficiently explain what is new compared to existing KM reviews, nor does it integrate the findings through a clear theoretical lens.

**Author response:** Thank you for this comment. We revised the Introduction to clarify the novelty of the study. The manuscript now explains that the review does not only identify KM factors, but also organizes them into an integrative conceptual framework. In addition, we added a new subsection, Section 4.3 Conceptual Framework, to synthesize the identified factors into broader conceptual categories and explain their contribution to KM effectiveness.

 2. The research question is broad, and the conceptual framework requires stronger theoretical justification.

**Author response: **Thank you for the suggestion. We revised the research question to make it more focused and aligned with the conceptual framework. We also strengthened the theoretical justification by explaining the process-based understanding of KM in the Literature Review and Discussion sections.

 3. The search strategy is narrow (single database and keyword), and the exclusion criteria, especially the exclusion of review articles, are weakly justified. These choices limit coverage and raise concerns about selection bias.

**Author response:** Thank you for highlighting this issue. We added further explanation in the Methods section to clarify that the use of Scopus as a single database, the keyword “Knowledge Management,” and the inclusion/exclusion criteria represent methodological limitations of this review. We also added a dedicated Limitations section acknowledging that these choices may limit coverage and introduce potential selection bias.

 4. The discussion and conclusions summarize findings, but lack deeper analytical synthesis, cross-study comparison, and explanation of relationships among factors.

**Author response:** Thank you for this valuable comment. We revised the Discussion by adding a conceptual framework section, cross-study comparison, and explanation of relationships among the identified factors. The Conclusion was also revised to emphasize the integrated interpretation of the findings rather than only summarizing individual variables.

# Reviewer report: Reviewer response for version 1

**DOI:** 10.5256/f1000research.182019.r409817

**Published:** 2025-09-19

**Authors:** Umair Zia

**Affiliations:** 1 Zhengzhou University, Zhengzhou, China

This study systematically reviews 44 articles between (2020–2024) using PRISMA to examine critical factors shaping knowledge management (KM) in business. The paper lacks methodological rigor, as the review process and criteria for selecting the 44 articles are not sufficiently detailed or justified, which undermines the credibility of the reseach findings.

Are the rationale for, and objectives of, the Systematic Review clearly stated?

Partly

Is the statistical analysis and its interpretation appropriate?

I cannot comment. A qualified statistician is required.

If this is a Living Systematic Review, is the ‘living’ method appropriate and is the search schedule clearly defined and justified? (‘Living Systematic Review’ or a variation of this term should be included in the title.)

Not applicable

Are sufficient details of the methods and analysis provided to allow replication by others?

No

Are the conclusions drawn adequately supported by the results presented in the review?

Partly

Reviewer Expertise:

Green, Knolwegde management. Organizational performance

I confirm that I have read this submission and believe that I have an appropriate level of expertise to state that I do not consider it to be of an acceptable scientific standard, for reasons outlined above.

# Response

**Published:** 2025-12-09

**Authors:** Muhammad Taufik Mardian

**Affiliations:** Faculty of Administrative Scie, Brawijaya University, Malang, East Java, Indonesia

Thank you for this valuable comment regarding the methodological rigour of the review. The Methods section has been revised to provide a clearer and more transparent account of the review process and article selection. In particular, a new Table 1 (“Search strategy and results”) has been added, summarising each screening stage, the corresponding inclusion criteria, and the number of records retained at each step, from initial identification to full-text eligibility. The description of the inclusion and exclusion criteria has also been refined to better justify the selection of the final 44 articles. These revisions are intended to strengthen the credibility and replicability of the review. We sincerely appreciate your constructive feedback and consideration, which have been very helpful in improving the methodological clarity and overall quality of our manuscript.

# Reviewer report: Reviewer response for version 1

**DOI:** 10.5256/f1000research.182019.r407295

**Published:** 2025-09-04

**Authors:** Harmon Chaniago, Yen Efawati

**Affiliations:** 1 Magister Management, Adhirajasa Reswara Sanjaya University, Bandung, JAWA BARAT, Indonesia; 2 Bandung State Polytechnic, Bandung, Indonesia

Title: Ok

 Abstract:

 The topic is clear, the research objectives are specific, and the methods are stated.

 Please add key findings and revise keywords for greater focus.

 Introduction:

 Overall, it's okay, but needs improvement:

 • Some sentences are too long; please make them more concise and avoid repetition.

 • Research gaps are still general and not clear enough. Please improve.

 • Formulate research objectives with only 2-3 main focuses.

 2. Literature review and Hypotheses:

 Generally, ok

 3. Methodology:

 Completed review standards (PRISMA/scoping).

 Needs to be completed/improved:

 • PRISMA flowchart is not detailed (numbers for each stage are missing). Please add a complete PRISMA flowchart with the number of articles at each selection stage.

 • Inclusion/exclusion criteria are still too general.

 • Provide a search strategy table (database, keywords, number of results).

 • Please consider using analysis software (VOSviewer, NVivo, R/Bibliometrix) to increase the credibility of this research.

 4. Results:

 The data has been presented in tables/figures, with a clear structure per category.

 Needs to be completed/improved:

 • The summary table is still a list, lacking analysis. Create a concise table with additional columns for "Country, Method, Key Findings."

 • The figure does not show the number of studies per category. Include numbers in the figure to clarify the distribution.

 5. Discussion

 Linking results to the literature and creating a framework.

 Needs to be completed/improved:

 • The discussion focuses on repeating results rather than analyzing causes. Please discuss factors contributing to differences in findings (e.g., developing vs. developed country contexts).

 • Theoretical contributions are unclear. Emphasize the novelty of the framework: how does it differ from previous models?

 • Add logical comparisons with other systematic reviews.

 6. Conclusion:

- Please separate theoretical, practical contributions, and future research directions. Use data-driven sentences.

7.  Others:

 Dear Authors, please use a different text color when submitting revisions to the article.

 Thank you for your effort

Are the rationale for, and objectives of, the Systematic Review clearly stated?

Partly

Is the statistical analysis and its interpretation appropriate?

Partly

If this is a Living Systematic Review, is the ‘living’ method appropriate and is the search schedule clearly defined and justified? (‘Living Systematic Review’ or a variation of this term should be included in the title.)

Partly

Are sufficient details of the methods and analysis provided to allow replication by others?

Yes

Are the conclusions drawn adequately supported by the results presented in the review?

Yes

Reviewer Expertise:

Organization Behaviour; Human Resource Management; Entrepreneurship; Business Strategy entrepreneurship, business strategy, small business, retail, innovation, digital business, and economic environment

We confirm that we have read this submission and believe that we have an appropriate level of expertise to confirm that it is of an acceptable scientific standard.

# Response

**Published:** 2025-12-09

**Authors:** Muhammad Taufik Mardian

**Affiliations:** Faculty of Administrative Scie, Brawijaya University, Malang, East Java, Indonesia

We thank the reviewer for the constructive and detailed feedback. The manuscript has been revised accordingly as outlined below.

**Title**

**Comment:** Title: Ok.

**Response:** No changes were made to the title.

**Abstract**

**Comment:** Please add key findings and revise keywords for greater focus.

**Response:** The Abstract has been revised to explicitly summarise the main findings of the review. The keywords have also been refined to focus more clearly on the core concepts and methods covered in the study.

**Introduction**

**Comment:** Some sentences are too long; please make them more concise and avoid repetition. Research gaps are still general and not clear enough; formulate research objectives with only 2–3 main focuses.

**Response:** The Introduction has been edited to remove repetitive and unnecessarily long sentences, making the narrative more concise and focused. The research gaps have been clarified and specified, and the research objectives have been reformulated.

**2. Literature Review**

**Comment:** Generally ok.

**Response:** No major structural changes were required.

**3. Methodology**

**Comment:** PRISMA flowchart is not detailed; please add numbers at each selection stage.

**Response:** The PRISMA flowchart (Figure 1) include the number of articles at each identification, screening, eligibility, and inclusion stage, in line with PRISMA reporting standards.

**Comment:** Inclusion/exclusion criteria are still too general.

**Response:** The description of the inclusion and exclusion criteria has been expanded and specified in greater detail, explaining conceptual, methodological, temporal, and language-related boundaries of the review.

**Comment:** Provide a search strategy table (database, keywords, number of results).

**Response:** A search strategy table has been added as **Table 1**.

**4. Results**

**Comment:** The summary table is still a list, lacking analysis.

**Response:** We have revised the summary of the included studies by adding a new Table 3, which restructures the information into more analytical columns to enhance clarity and readability.

**5. Discussion**

**Comment:** The discussion still repeats results with limited causal analysis, unclear theoretical contributions and framework novelty, and insufficient comparison with previous systematic reviews.

**Response:** We have clarified the theoretical contributions by adding **Figure 3**, which presents the proposed conceptual framework as the main theoretical contribution of this review. The figure highlights how the model extends previous frameworks and can be used as a basis for future empirical research.

**6. Conclusion**

**Comment:** Please separate theoretical, practical contributions, and future research directions, using data-driven sentences.

**Response:** The final section has been reorganised into two subsections, Conclusion and Recommendations.

 We sincerely thank the reviewer for the insightful comments, which have significantly improved the clarity, rigour, and contribution of our manuscript.
